# Writing Errors and Anosognosia in Amyotrophic Lateral Sclerosis with Dementia

**DOI:** 10.1155/2008/814846

**Published:** 2008-07-15

**Authors:** Hiroo Ichikawa, Shinichi Koyama, Hideki Ohno, Kenji Ishihara, Kiyomi Nagumo, Mitsuru Kawamura

**Affiliations:** ^1^Department of NeurologyShowa University School of Medicine 1-5-8 HatanodaiShinagawa-kuTokyo 142-8666Japan; ^2^Division of Clinical ElectrophysiologyDepartment of NeurologyUniversity of IowaIA 52241USA

**Keywords:** Amyotrophic lateral sclerosis, dementia, writing error, paragraphia, anosognosia

## Abstract

Amyotrophic lateral sclerosis (ALS) with dementia (ALS-D) is known to exhibit characteristics of frontotemporal dementia. However, in clinical situations, it is often difficult to evaluate their cognitive functions because of impaired voluntary speech and physical disabilities. In order to identify characteristic and diagnostic cognitive symptoms of relatively advanced ALS-D patients, we retrospectively reviewed the clinical features of seven cases of clinically definitive ALS who had dementia, impaired voluntary speech, and physical disability. Their medical records showed that six out of seven patients made writing errors, and all of the patients demonstrated anosognosia. The writing errors consisted of paragraphia such as substitution, omission, or syntactic errors with individual differences in error types. Dissociation between kana and kanji were also observed. Anosognosia was evaluated by a self-rating scale with which the patients and the medical staff evaluated the patient's physical ability; the results indicated a large discrepancy between the evaluation by the patients and the medical staff. We emphasize that aphasic writing errors have been underestimated, particularly in ALS-D patients with impaired voluntary speech. We also reported that anosognosia was the most important and quantifiable symptom in ALS-D. The relationship between writing errors and anosognosia should be investigated further.

## 1. Introduction

Motor neuron disease (MND) or more specifically, amyotrophic lateral sclerosis (ALS) accompanying dementia (MND-D/ALS-D) is known to be associated with characteristics of frontotemporal dementia (FTD) [6,19,23,26,28,30,36,42]. Clinically, FTD produces early changes in behavior and executive function with relatively preserved memory and praxis [20,27]. The recent consensus of the clinical diagnostic criteria for FTD postulated loss of insight as a core diagnostic symptom, a characteristic of the clinical syndrome of frontotemporal lobar degeneration (FTLD), including other subtypes of non-fluent progressive aphasia (NFPA) and semantic dementia (SD) [20,27]. Recent studies have shown that ubiquitin-positive, tau-negative inclusions can be a pathological hallmark of MND-D/ALS-D; considering those neurophathological findings, MND-D/ALS-D has been included under the spectrum of FTD without Pick bodies [18, 29,40,43]. However, the frequency of overlapping ALS/MND with dementia and/or language disturbance varies across reports [4,19,30,31,36]; dissociation between clinical and pathological diagnosis have been reported, such as cases of pathologically defined MND-D/ALS-D clinically lacking dementia [25,43]. Thus, the diagnosis of MND-D/ALS-D remains difficult. In clinical situations, cognitive functions are often difficult to evaluate because of impaired voluntary speech and/or physical disabilities. Thus, we attempted to evaluate the common, characteristic, and diagnostic

ISSN 0953-4180/08/$17.00 © 2008 – IOS Press and the authors. All rights reserved symptoms of MND-D/ALS-D patients with impaired voluntary speech and/or physical disabilities by reevaluating their medical records and examining their writing problems.

## 2. Methods

### 2.1. Patient selection and profiles

We recruited seven patients with ALS-D by reviewing the medical records of 121 consecutive MND/ALS patients admitted to our hospital from 1989 to 2004. Based on daily behavior evaluations, the seven patients were diagnosed with dementia. Neuropsychological examinations and neuroradiological findings were also considered. All seven patients fulfilled the clinical and electrophysiological criteria for clinically definite ALS according to the Revised Criteria for the Diagnosis of Amyotrophic Lateral Sclerosis by the World Federation of Neurology Research Group on Motor Neuron Disease: El Escorial revised [41]. Two of the patients were confirmed to have ALS based on their neuropathological findings.

Table 1 shows a summary of the seven patients – four male patients and three female patients. The age of onset ranged from 49 to 80 years, and the average was 63. All the patients were right-handed. Six patients had bulbar onset, while one patient had limb-onset. In three patients, dementia had occurred prior to the motor disorders, whereas in the remaining four patients, the motor disorders occurred prior to dementia. The duration of dementia varied from 2 to 113 months, and that of motor disorders varied from 8 to 45 months with an average of 23 months (in cases wherein a respirator was used, the duration before the use of the respirator has been shown).

### 2.2. Evaluation of dementia and writing

We retrospectively reevaluated the clinical features, particularly the daily behavior, of the patients. In addition, we also reviewed their writing. Patients with writing problems were evaluated for their characteristic features.

We also reviewed some neuropsychological tests mainly obtained from the medical records. The neuropsychological examinations included the Hasegawa dementia scale (HDS), HDS-Revised (HDS-R), Mini Mental State Examination (MMSE), Wechsler Adult Intellectual Scale-Revised (WAIS-R), Kana Pick-up Test and Wisconsin Card Sorting Test (WCST). However, six out of seven patients could not complete all the tests due to their physical disabilities. HDS, HDS-R, and Kana Pick-up Test have been developed in Japan as scales for dementia. HDS-R is a simple test similar to MMSE and is supposed to correlate well with MMSE [13].

In addition, we evaluated the radiological findings of X-ray computed tomography (CT), magnetic resonance imaging (MRI), and single photon emission CT (SPECT).

### 2.3. Self-rating scale for anosognosia

To objectively evaluate awareness of illness, we applied a quantitative self-rating scale for anosognosia (Deckel et al. (1966) [8]) to two patients (Table 2). Using 5-point scale, the patients evaluated his/her own activities of daily living (ability to walk, ability to speak fluently, etc.). The medical staff made an additional evaluation based on this scale, and the scores recorded by the patient and the average of the scores recorded by two medical staff members were compared. The gap between the score would indicate the degree of anosognosia (maximum gap = 32).

## 3. Results

### 3.1. Clinical features

The clinical time-courses of the three patients with dementia prior to ALS (patients 1, 2, and 3) and those of the four patients who developed ALS prior to dementia (patients 4, 5, 6, and 7) are summarized in Table 3. In patient 1, dementia presented with symptoms such as restlessness, extravagance, and errors with simple calculations; the patient was first diagnosed with Pick's disease. In patient 2, dementia presented with symptoms such as reduced volition, reduction of speech, and social withdrawal. A psychiatrist diagnosed the patient with depression at an early stage of the disease. In patient 3, dementia presented with symptoms such as excessive attachment for money, excessive suspicion and vigilance, and compulsive behavior. The patient was first diagnosed as having Pick's disease. In patients 1–3, dementia was first noted 13, 29, and 90 months prior to the appearance of motor symptoms, respectively. On the other hand, in patients 4–7, the motor symptoms were first noted 9, 40, 14, and 16 months prior to dementia, respectively. Their neuropsychological prob-

Table 1
Summary of the patients with ALS-D

| Patient | Age** (y) /Gender | Dominant hand | Motor symptoms | Type of ALS | Initial symptoms | Duration of illness (months) Dementia | ALS |
|---|---|---|---|---|---|---|---|
| 1 | 49/M | Right | L > M > B | Limb-onset | Dementia | 21 | 8 |
| 2* | 59/F | Right | B > L < U | Bulbar | Dementia | 30 | 11 |
| 3 | 60/M | Right | B > L $\geqq$ U | Bulbar | Dementia | 113 | 23 |
| 4 | 67/M | Right | B > L > U | Bulbar | ALS | 19 | 28*** |
| 5* | 54/F | Right | B > L > U | Bulbar | ALS | 5 | 45*** |
| 6 | 73/F | Right | B > L > U | Bulbar | ALS | 2 | 16*** |
| 7 | 80/M | Right | B > L > U | Bulbar | ALS | 12 | 28 |

Average age: 63 year-old; male (M) 4 patients; Female (F) 3 patients; Average duration of motor symptoms: 23 months.
*Autopsied cases defined as ALS-D; **age at the onset; ***Duration until artificial ventilation
Abbrenviations: L, lower motor neuron sign; U, upper motor neuron sign; B, bulbar sign.

Table 2
Anosognosia scale

| | | | | |
|---|---|---|---|---|
| 1. Walk | | | | |
| Very impaired | Below average | Average | Above average | Excellent |
| 2. Accurately and quickly use hands and fingers | | | | |
| Very impaired | Below average | Average | Above average | Excellent |
| 3. Speak clearly | | | | |
| Very impaired | Below average | Average | Above average | Excellent |
| 4. Remember | | | | |
| Very impaired | Below average | Average | Above average | Excellent |
| 5. Concentrate and attend | | | | |
| Very impaired | Below average | Average | Above average | Excellent |
| 6. Sit “still and quietly | | | | |
| Very impaired | Below average | Average | Above average | Excellent |
| 7. Say the word you are thinking of | | | | |
| Very impaired | Below average | Average | Above average | Excellent |
| 8. Control your emotions | | | | |
| Very impaired | Below average | Average | Above average | Excellent |

Quoted from Deckel et al. (1996) [8].

lems were observed to be reduced volition and apathy (patient 4), restlessness and disinhibition (patient 5), repetition of the same story and stubbornness (patient 6), and unconcern with the illness (patient 7).

The neuropsychological symptoms observed in the patients are summarized in Table 4. Frequently noted symptoms were diminished spontaneous speech, apathy, reduced volition, disinhibition, attention deficits, restlessness, and going-away behavior. Memory disorder, errors with calculations, perseveration, and imitation behavior were also occasionally seen. In addition to the above symptoms, unawareness or unconcern for illness was noted in all patients by the medical staff.

### 3.2. *Writing*

The characteristics of writing errors are summarized in Table 5. Although established written tests were not performed, writing errors were evident in all patients, except patient 3 from whom a writing sample was not obtained. These problems were first noticed through communication by writing. Meanwhile, aphasic speech had not been noted in any patients before evaluating their writing. Five patients (patient 2, 4, 5, 6, and 7) had almost lost the ability to speak, rendering evaluation of spoken language impossible. Patient 1 was able to speak, but no aphasic speech was noted.

The Japanese system of writing consists of two types of letters – kana (phonogram) and kanji (ideogram). Kana represents a spoken syllable or “mora,” and the sound-to-script correlation is strictly one-to-one. On the other hand, each kanji, which is not used in spoken language, conveys a particular meaning and has a few different pronunciations. We also observed different elements of errors and some dissociation between errors in kana and kanji letters, in each case.

Figure 1 shows some writing samples of the patients in Japanese. Spoken language was normal in patient 1; however, paragraphia was seen in kanji, when the patient was instructed to explain illustrations of daily scenes: the phonologic paragraphia in kanji referring to the substitution of another kanji that has the same pho-

Table 3
Summary of patients' clinical time courses

| Patients preceeded by dementia | Initial symptpms of dementia | —- | Interval (months) | —- | Initial motor symptoms |
|---|---|---|---|---|---|
| 1 | Restlessness; Extravagance; Errors with simple calculations<br>Initial diagnosis was Pick's disease | | 13 | | Fasciculation of upper limbs |
| 2 | Reduced voliton;Reduction of speech; Social withdrawal<br>Initial diagnosis was depression at Psychiatry | | 29 | | Bulbar symptoms |
| 3 | Excessive attchnment for money; Excessive suspicion and vigilance; Compulsive behavior<br>Initial diagnosis was Pick's disease | | 90 | | Bulbar symptoms |
| **Patients preceeded by ALS** | **Initial motor symptoms** | **—-** | **Interval (months)** | **—-** | **Initial symptoms of dementia** |
| 4 | Bulbar symptoms | | 9 | | Reduced volition; Apathy |
| 5 | Bulbar symptoms | | 40 | | Restlessness; Disinhibition |
| 6 | Bulbar symptoms | | 14 | | Repetition of the same story; stubbornness |
| 7 | Bulbar symptoms | | 16 | | Unconcern with the illness |

Table 4
Summary of neuropsychological features

| Symptoms | Patients | | | | | | |
|---|---|---|---|---|---|---|---|
| | 1 | 2 | 3 | 4 | 5 | 6 | 7 |
| Diminished spontaneous speech | | + | | + | + | + | + |
| Apathy or reduced volition | | + | | + | + | | + |
| Disinhibition | + | + | + | | + | + | |
| Attention deficits | + | + | + | | + | + | + |
| Restlessnes | + | + | + | | + | + | |
| Going away behavior | + | + | + | | | + | |
| Acalculia or loss of memory | + | | | | | | + |
| Perseveration | + | | | + | | + | |
| Imitation Behavior | | + | | | | + | |
| Unawareness or unconcern for illness | + | + | + | + | + | + | + |

netic value as the correct answer [e.g., "患者 (kan-ja)" (correct writing) meaning patient => "感者 (kan-ja)" (patient's writing)] and the morphologic paragraphia in kanji referring to the substitution of another kanji that has visual resemblance to the correct answer [e.g., "受 (u)" (correct answer) => "愛 (ai)" (patient's answer)] (Fig. 1-A). Patient 5 who was almost mute also exhibited paragraphia in kanji, when she answered our questions by writing: the phonologic and morphologic paragraphia [e.g., "黄門 (kou-mon)" (correct writing) => "公園 (kou-en)" (patient's writing) meaning park; "元気" (correct writing) meaning fine => "先気" (patient's writing)], (Fig. 1-B). These writing errors appeared predominant in kanji.

Patient 4 who was already mute provided his medical history written by himself, which contained literal omissions of kana [e.g., "ひどくなった" (correct writing) meaning worsened => "ひどった" (patient's writing), "くっついている" (correct writing) meaning attached => "くついる" (patient's writing)]. He was however able to write complex kanji letters (Fig. 1-E). Patient 6 who was already mute exhibited the substitution of kana prominent in postpositions [e.g., "に", "の", and "で" representing prepositions "to", "of", and "by", respectively] in her spontaneous writing, suggesting grammatical errors (Fig. 1-F). When she was tested with the HDS-R, she exhibited substitution and omission of kana [e.g., "びょういん" (correct writing) meaning hospital => "びよでん" (patient's writing)], and perseveration (Fig. 1-G). These writing errors appeared predominant in kana.

As shown in Table 5 and Fig. 1, paragraphia was a consistent feature of the writing errors, but its characteristics varied in cases: variation in appearance of substitution, omission and syntactic errors and some dissociation in errors between kanji and kana.

### 3.3. *Self-rating scale for anosognosia*

In our retrospective review, anosognosia was noted in all patients. A more detailed analysis of the quan-

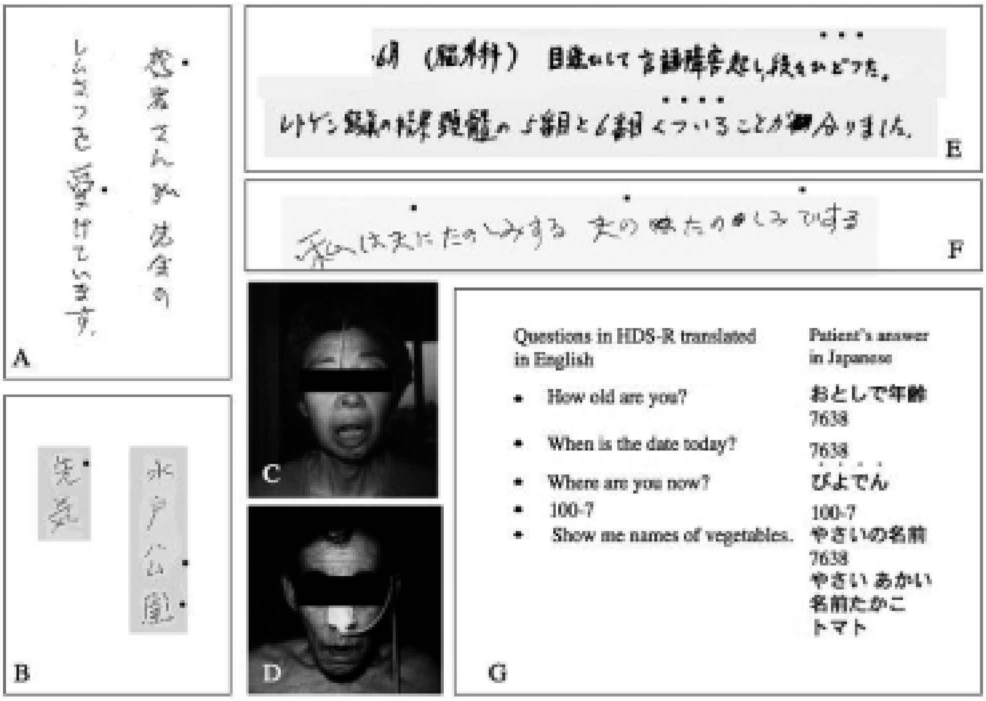

Fig. 1. Samples of writing errors in Japanese: A. Paragraphia in kanji observed in patient 1, when instructed to explain illustrations drawing daily scenes. One is the phonologic paragraphia of kanji [“患者 (kan-ja)” (correct writing) meaning patient => “ (kan-ja)” (patient's writing)] and the other is morphologic paragraphia of kanji [“受 (u)” (correct writing) meaning receive => “愛 (ai)” (patient's writing) meaning love]. Writing of kana letters was well preserved. B. Paragraphia in Kanji observed in the patient 5. When we asked her “ what TV show are you watching?”, she wrote “水戸公園 (mi-to-kou-en) in spite that a correct answer is “水戸黄門 (mi-to-kou-mon)” which is the title of her best favorite TV show. When we asked “How are you?”, she wrote “先気 (sen-ki)” to the question in spite that a correct answer is “元気 (gen-ki)” meaning “fine”. These represent phonologic and morphologic paragraphia in kanji. C. D. Facial appearance of patients 4 and 5. Both patients have a transnasal tube inserted due to severe bulbar symptoms. E. Paragraphia in kana observed in a medical history written by Patient 4. The omission of kana was particularly remarkable in his writing [“ひどくなった (hi-do-ku-na-tta)” (correct text) meaning worsened => “ひどつた (hi-do-tsu-ta)” (patient's version); “くっついている (ku-ttsu-i-te-i-ru)” (correct text) meaning attached => “くついる (ku-tsu-i-ru)” (patient's version)]. Some lack of postpositional particles was also observed. However, he was capable of writing difficult kanji letters, and writing in kanji was preserved. F. Paragraphia observed in patient 6, during communication by means of writing by the bedside in the hospital. Incorrect use and lack of postpositional particles in kana letters were particularly remarkable. For instance, “に”, “の”, and “で” approximately correspond to the English prepositions “to”, “of”, and “by”, respectively [“私は夫をたのしみにする(watashi wa o-tto o ta-no-shi-mi ni su-ru), (夫をたのしみにするo-tto o ta-no-shi-mi ni su-ru)” (correct text) => “私は夫にたのしみする (wa-ta-shi wa o-tto ni ta-no-shi-mi su-ru), 夫のたのしみでする (o-tto no ta-no-shi-mi de su-ru)” (patient's version)]. G. The writing sample of patient 6 is her response in the HDS-R test. When questioned “how old are you?”, she wrote “お年で年齢 (o-to-shi-de-nen-rei),” which roughly translated to “age and age,” and then wrote her telephone number—7638. When asked “what is the date today?”, she wrote 7638 again. When she was asked “where are you?”, she wrote “びよでん (bi-yo-den)” instead of the correct answer “びょういん (byo-u-in),” which means hospital. When we instructed her to calculate “100-7”, she wrote “100-7”. Thus she exhibited perseveration, substitution and omission of kana letters.

titative self-rating scale for anosognosia (Deckel et al., 1996) [8] on two patients (patients 6 and 7) revealed a large gap in the ratings as evaluated by the patients and those recorded by the medical staff. The gap between the scores indicates the degree of anosognosia (maximum gap = 32). In patients 6 and 7, the gaps between the patient and the medical staff were 8 and 18 points, respectively (Fig. 2). Interestingly, even though their upper limbs did not show fluid/smooth movement at that time, they denied acknowledging their disability and insisted through gestures that they could move their fingers easily. We performed the same test on three patients with ALS without dementia, and none of them showed these gaps.

### 3.4. Neuropsychological tests

Due to physical disabilities, neuropsychological tests were performed only in five patients (Table 6). Of those, only patients 5 and 6 were diagnosed with dementia by HDS or HDS-R tests at their first examination. Although patient 1 was diagnosed as not having dementia by HDS or HDS-R, his scores in the WAIS-R

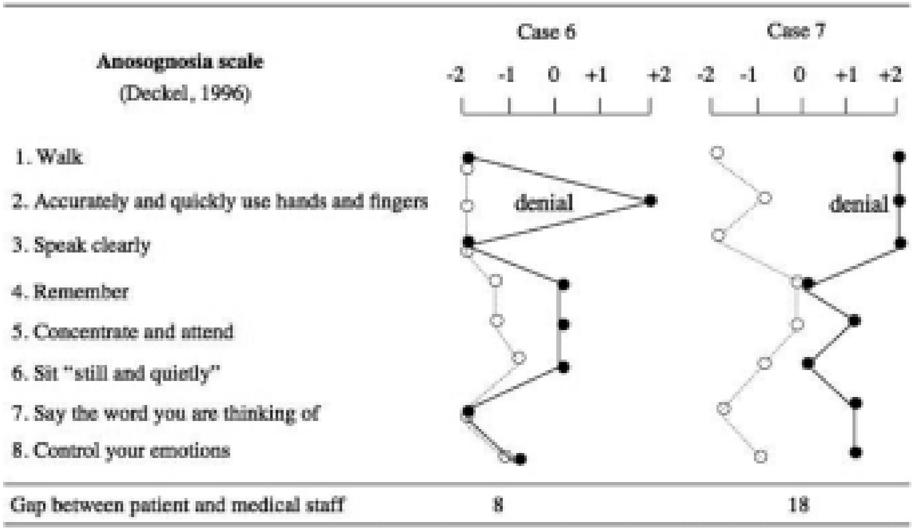

Fig. 2. Results of the anosognosia scale applied in two patients; The results of the anosognosia scale that was used in the case of two patients (patients 5 and 6) are shown. The score of a patient's evaluation is indicated by a closed circle, and the averaged values as evaluated by the two medical staff members are indicated using open circles. The discrepancy in the score is prominent. The average of the scores is 13 $\pm$ 7.07 (standard deviation: SD). On the other hand, the average of scores in three patients with ALS without dementia was 0.17 $\pm$ 0.29 (SD).

test, WCST, and Kana Pick-Up Test were poor. Patient 3 performed poorly in the Kana Pick-Up Test but was diagnosed as not having dementia by the HDS-R and WAIS-R tests.

### *3.5. Radiological findings*

In all patients, CT and MRI findings revealed bilateral frontotemporal atrophy without vascular lesions. SPECT imaging showed reduced uptake in the bilateral frontotemporal lobes in all patients and was seen more robustly in the left hemisphere in three patients (patients 3, 6, and 7).

## 4. Discussion

MND-D/ALS-D has been found to be closely associated with FTD [6,19,23,26,28,30,36,42]. The frequent neuropsychological symptoms were reduced spontaneous speech, apathy, reduced volition, disinhibition, attention deficits, restlessness, and going-away behavior; these symptoms are characteristic features of FTD [20,27]. The features of ALS in this study, however, were mostly observed to be of the bulbar-type, as generally reported [6,19,23,26,28,30,36,42]. Three patients developed dementia before motor symptoms, and one of these patients was atypical in that he developed bulbar ALS after a 7-year history of dementia. We found an autopsy report similar to this atypical case, wherein the deceased had developed ALS after a long period following its onset [39]. On the other hand, four patients developed motor symptoms before dementia. Although dementia is frequently reported to occur before ALS symptoms appear, observation contradicting this has also been reported; for example, Portet et al. reported that cognitive decline developed in bulbar MND/ALS at a high frequency (48%) and that dementia always occurred after motor neuron sign and is not clinically evident during first six months of disease onset [30]. They also mentioned that the onset of dementia is often difficult to define and the first signs are usually unrecognized or misdiagnosed as mood disorders [30]. We believe that temporal order between dementia and MND/ALS is more flexible than commonly considered.

Writing errors, which were frequently observed in our study, are particularly remarkable. Among the six patients whose writing samples were available, all exhibited obvious/evident writing errors, although up till then no aphasic syndrome had been noted. A common feature of writing errors is paragraphia, which is characteristic and indicative of an aphasic condition. Interestingly, in the present study, the features of paragraphia varied across cases. In addition, we observed some dissociation between the errors in kana and kanji, although we could not conclusively demonstrate the dominance in errors between the two because the task demands in these tests was not fully controlled. As

Table 5
Summary of the writing problems and the background at that time

| Symptoms | | Patients | | | | | | |
|---|---|---|---|---|---|---|---|---|
| Type of ALS | | 1 | 2 | 3 | 4 | 5 | 6 | 7 |
| Voluntary speech | | Limb-onset | Bulbar | Bulbar | Bulbar | Bulbar | Bulbar | Bulbar |
| History of aphasic speech | | Preserved | Mute | Preserved | Mute | Almost mute* | Mute | Almost mute* |
| | | —- | —- | —- | —- | —- | —- | —- |
| Wrigting errors | | + | + | N.A. | + | + | + | + |
| Paragraphia | | + | + | | + | + | + | + |
| Kanji | Phonologic substitution | + | + | | | + | | |
| | Morphologic substitution | + | + | | | + | | |
| Kana | Substitution | | + | | + | | + | |
| | Omission | | + | | + | | + | + |
| | Syntactic error | | | | + | | + | |
| Perseveration | | + | | | + | | + | |

*They were incapable of comprehensible speech.
N.A.: not available. Writing samples could not be obtained from patient 3.
Phonologic substitution in kanji refers to the substitution with another kanji that sounds the same as the correct answer. [e.g時計 (to-kei) meaning watch → 戸計 (to-kei), written by Patient 2]; Morphologic substitution in kanji refers to the substitution with another kanji that visually resembles the correct answer. [e.g. 墓 (haka) meaning grave → 幕 (maku) meaning curtain, written by Patient 2]; Substitution in kana refers to instances where one or more kana letters that constitute a kana word were substituted for other kanas [e.g.はさみ (ha-sa-mi) meaning scissors → はさめ (ha-sa-me), written by Patient 2]; Omission in kana refers to instances where one or more kana letters that constitute a kana word were omitted. [e.g. えんぴつ (e-n-pi-tsu) meaning pencil → えぴつ (e-pi-tsu), written by Patients 2 and 7] ; Syntactic error refers to wrong use or lack of kana letters exclusively for postpositional particles.

Table 6
Results of neuropsychological examinations

| | | 1 | 3 | 5 | 6 | 7 |
|---|---|---|---|---|---|---|
| HDS | | 30/32.5 | | 16.5/32,5 | | |
| HDS-R | | 26/30 | 29 /30 (→*1 0/30) | | 0/30 | 21/30 |
| MMSE | | 19/30 | | | | |
| WAIS-R | VIQ | 65 | 89 | | | |
| | PIQ | 67 | 109 | | | |
| | FIQ | 62 | 97 | | | |
| Kana Pick-up Test | | 18/60 | 23/60 | | | |
| WCST | Categories | 1/6 | | | | |
| | Preservation errors | 62.5% | | | | |

Examinations were performed once during the patients' clinical courses, except for patient 3 who underwent a follow-up examination (→*). Patients 5, 6 and 7 were not able to speak at the examination, which was a written test. Patients 2 and 4 were not able to complete any examination.
A score of 21.5 or less for HDS and 20 or less for HDS-R suggested dementia.

described previously, the Japanese system of writing consists of two types of letters – kana (phonogram) and kanji (ideogram), and dissociation of impairment between these two kinds of letters has been reported [15, 17,34]. Although the precise topographical correlation for such conditions remains unclear, some authors have prompted a hypothesis suggesting that the mechanisms involved in processing kanji and kana are different [15, 17,34]. Iwata et al. [15] postulated that Wernicke's area yields different pathways to the left frontal motor areas. In a report that described frontal pure agraphia in kanji or kana, two different pathways of phonologic and morphologic routes, traveling from the left temporal to the left frontal lobe, were postulated [34]. Considering these descriptions, left frontotemporal impairment is supposed to be responsible for the writing errors observed in our study, and the different features of writing errors might suggest rather selective impairment of the left frontotemporal lobe in ALS-D. However, all these reports are mostly based on cerebrovascular diseases. On the other hand, such reports in ALS-D are extremely rare, but there are two case reports [14,16] referring to a similar observation, that describe either the omission of kana letters or paragraphia of kanji, respectively.

With regard to syntax writing errors in ALS-D, Ferguson et al. [10] have already reported two cases of bulbar-onset ALS that presents along with syntactic writing errors. Later in 1990s, some authors [9,31] also mentioned syntactic writing errors in MND patients, in their detailed report on language deficits in MND

patients. In addition, Bak et al. [1] recently reported that six patients with MND exhibited impaired syntactic comprehension and possessed selective pathological changes in Broadmann area 44 (Broca's area) and 45. The findings of a several neuropsychological studies and recent neuroimaging studies also suggest that Broca's area plays an important role in the processing of syntax [3,11,12,37].

Although patients with MND/ALS presenting with progressive aphasic syndrome as a main feature are accumulated [1,4,5,9,31,38], such cases have yet been considered as a unique and unusual condition. It is still controversial whether typical MND-D/ALS-D and MND/ALS with aphasia form separate disease entities or can be viewed as extreme forms of a cognitive deficit characteristic of MND/ALS in general [2,4,5,9,31,38]. From our study, we speculate that aphasic conditions are more common in MND-D/ALS-D than previously considered, and aphasia might be simply masked by reduced voluntary speech and bulbar symptoms, as postulated somewhere [31].

Anosognosia is also a remarkable cognitive symptom. It was the most consistently noted symptom as loss of insight – postulated as a core diagnostic criterion for FTD [20,27] – was frequently reported in this study. Rankin et al. [32] recently examined patients with FTD and Alzheimer's disease (AD) by using a self-rating scale for personality (the Interpersonal Adjective Scale) to assess the loss of insight, and they reported that the gap between the patients and first-degree relative informants was greatest in FTD. On the other hand, Deckel et al. [8] used a self-rating scale (anosognosia scale) that they developed to quantitatively assess anosognosia or denial of illness in Huntington's disease (HD). This scale consisted of eight questions pertaining to the ability to perform daily living activities, and the rating for the patient's abilities was evaluated on a 5-point scale by the patients and additionally by medical staff members who were well-acquainted with them [8]. The difference between the scores evaluated by the patient and medical staff was computed and was considered to indicate the degree of anosognosia (maximum difference = 32 points) [8].

The anosognosia scale was applied to two of our patients with severe physical inabilities, and the gaps were 8 and 18 points, respectively. The gaps were greater than those reported by Deckel et al., which averaged 5.81 in the HD group and 1.2 in the normal control group [8]. Notably, both our patients denied their physical inabilities. Thus, the anosognosia scale could be useful for the quantitative and objective evaluation of anosognosia in MND-D/ALS-D patients. In addition, this scale is fairly simple to be performed within a few minutes and is therefore suitable for physically disabled patients.

Clinical and neuroradiological studies [7,22,33,35] reveal that anosognosia has been associated with impairments of the right frontal lobe and frontal executive function. In addition, the pathological study on AD by Marshall et al. [21] showed that the density of amyloid plaques in the right prosubiculum (transitional zone between the subiculum and the adjacent CA1 region of the hippocampus) correlated with anosognosia for the illness. Interestingly, the pathological changes in the same transitional zone between the subiculum and CA1 are believed to be common and occur early in MND-D/ALS-D [24,40]; they may be related to the anosognosia commonly seen in MND-D/ALS-D patients. Although the precise pathophysiological mechanism for anosognosia remains unclear, Marshall et al. [21] speculated that it was attributable to the dysfunction of the neuronal network connecting the prosubiculum, amygdala, entholinal cortex, and cingulated gyrus.

Although the relationship between writing errors and anosognosia is not clear, we speculate that writing errors indicate the aphasic condition derived from the left hemispheric lesion, and the right hemispheric lesion is responsible for anosognosia. We consider that typical ALS-D involves the bilateral frontotemporal lobes, but symptoms from the left hemispheric lesion are supposed to be easily masked by bulbar palsy and simply translated as reduced verbal output or mutism. In other words, only some patients with selective left hemispheric impairment and preserved articulation might present with a clinically typical progressive aphasic syndrome. In fact, although SPECT imaging showed left-side dominant reduced uptake in three patients, the bilateral frontotemporal lobe was involved in all patients.

In conclusion, our study suggested that writing errors and anosognosia would be common, characteristic, and diagnostic symptoms in relatively advanced ALS-D patients. Although the relationship between writing errors and anosognosia remained unclear, one possible hypothesis is that writing errors probably occurs due to the left hemisphere lesion and that anosognosia probably occurs due to the right hemisphere lesion. Further studies on linguistic aspects and the relationship between writing errors and anosognosia would be necessary.

## Acknowledgements

Our study was supported by JSPS, CREST, a Grant-in-Aid for Scientific Research on Priority Areas-< System study on higher-order brain functions >-from the Ministry of Education, Culture, Sports, Science and Technology of Japan (17022035, 18020027). It was also supported in part by a Showa University Grant-in-Aid for Innovative Collaborative Research Projects and a Special Research Grant-in-Aid for the Development of Characteristic Education from the Japanese Ministry of Education, Culture, Sports, Science, and Technology.
